# Analgesic Administration for Patients with Renal Colic in the Emergency Department Before and After Implementation of an Opioid Reduction Initiative

**DOI:** 10.5811/westjem.2018.9.38875

**Published:** 2018-10-18

**Authors:** Sergey Motov, Jefferson Drapkin, Mahlaqa Butt, Andrew Thorson, Antonios Likourezos, Peter Flom, John Marshall

**Affiliations:** * Maimonides Medical Center, Department of Emergency Medicine, Brooklyn, New York; † Peter Flom Consulting, New York, New York

## Abstract

**Introduction:**

We aimed to evaluate the patterns of analgesic prescribing for emergency department (ED) patients suffering from pain of renal colic before, during, and after implementation of an opioid reduction initiative. We hypothesized that this initiative based on the concept of channels/enzymes/receptors-targeted analgesia would result in overall decrease in opioid utilization in the ED and at discharge.

**Methods:**

We performed a retrospective analysis of ED electronic medical record of patients presenting with renal colic who received analgesics in the ED and at discharge over a five-year period. Patients were divided into three groups based on the following periods: 2012–2014 (pre-implementation phase); 2014–2015 (implementation phase); and 2015–2017 (post-implementation).

**Results:**

A total of 4,490 patients presented to the ED with renal colic over a five-year study period. Analgesics were administered to 3,793 ED patients of whom 1,704 received opioids and 2,675 received non-opioid analgesics. A total of 3,533 ED patients received a prescription for analgesic(s) upon discharge from the ED: 2,692 patients received opioids, and 2,228 received non-opioids. We observed a 12.7% overall decrease from the pre-implementation to post-implementation time period in opioid prescribing in the ED and a 25.5% decrease in opioid prescribing at discharge, which translated into 432 and 768 fewer patients receiving opioids, respectively.

**Conclusion:**

Implementation of an opioid-reduction initiative based on patient-specific, pain syndrome-targeted opioid alternative protocols resulted in a reduction in opioid administration in the ED by 12.7% and at prescriptions at discharge by 25.5%. Adoption of similar ED initiatives nationwide has the potential to foster effective non-opioid analgesic practices for ED patients presenting with renal colic and to reduce physicians’ reliance on administering and prescribing opioids.

## INTRODUCTION

The United States (U.S.) is in the midst of an opioid epidemic related to prescription opioids that has affected the lives of hundreds of thousands of people and their families. The uncontrolled prescribing of opioid analgesics in the 1990s resulted in collateral damage in the form of abuse, diversion, misuse, and development of opioid use disorder.^1^–^5^ Between 1999 and 2010, the rate of opioid prescribing increased by 700%.^1^,^2^ In 2012 alone, healthcare providers wrote 259 million opioid prescriptions, an amount sufficient to supply every American adult with a bottle of opioid pills.^3^–^5^ In 2014, 10.3 million persons reported using prescription opioids non-medically.^2^–^3^ More importantly, this massive escalation of prescription opioid use led to a 200% increase in mortality related to unintentional opioid overdose between 2000 and 2014.^6^–^13^ In fact, between 2013 and 2014 alone, opioid-related deaths in the U.S. increased 14%, from 7.9 to 9.0 per 100,000 population.^6^, ^7^ More recently, about 66% of approximately 64,000 drug overdose deaths in 2016 involved an opioid, which translates to an average of 115 Americans dying every day from an opioid overdose.^8^

This alarming rise in the rates of opioid abuse and death also reflects parallel increases in the rates of addiction and death resulting from the substitution of heroin for prescription opioids.^5^,^14^ Hospitalizations related to opioid misuse and dependence have also increased dramatically, with the rate of adult hospital-inpatient stays per 100,000 population nearly doubling between 2000 and 2012.^9^,^13^,^15^ This public health crisis calls for immediate interventions on behalf of all healthcare providers to identify safe and effective ways to control pain. One such intervention is implementation of opioid-reduction protocols that emphasize use of non-opioid analgesic modalities.

Despite both increases in emergency department (ED) visits and rising rates of opioid prescribing^16^–^18^ emergency physicians contributed less than 5% of total opioid prescriptions nationally (12.5 million prescriptions in 2012). In addition, emergency medicine (EM) as a specialty demonstrated the largest decrease in opioid prescribing rates (8.7% from 2007 to 2012) of all the medical specialties.^19^,^20^ However, even a short course of an opioid analgesic after discharge from the ED can lead to long-term (after one year) opioid use in up to 13% of opioid-naive patients.^21^,^22^ Similarly, prescriptions for opioid analgesics at discharge from the ED by “high-intensity prescribers” further augment this risk.^21^–^23^ It is prudent for physicians to consider non-opioid analgesic modalities in the ED and at discharge and resort to opioids only when the benefits of short-term therapies outweigh the risks of opioid-related adverse effects and/or non-opioid therapies fail to provide acceptable analgesia.

We aimed to evaluate the patterns of analgesic prescribing for ED patients suffering from pain of renal colic before and after implementation of an opioid-reduction initiative. We hypothesized that implementation of such initiatives that promulgate a patient-specific, pain syndrome-targeted approach for non-opioid analgesic modalities would result in overall decrease in opioid utilization for these patients in the ED and at discharge.

## METHODS

### Study Design and Setting

We performed a five-year retrospective analysis of all ED patients presenting with renal colic and receiving analgesics in the ED and at discharge by using the ED electronic medical record (EMR) (Allscripts™). We based the design and implementation of an opioid reduction initiative in our ED on the concept of channels/enzymes/receptors-targeted analgesia (CERTA) that focuses on patient-specific, pain syndrome-targeted pain control for a variety of acute and chronic painful conditions in the ED (Appendix 1).^24^,^25^ The CERTA approach promotes combinations of non-opioid analgesics as first-line treatment modalities when feasible and employs opioids judiciously and predominantly as a rescue. A pilot study of non-opioid analgesic administration conducted in our ED prior to implementation of the opioid reduction initiative demonstrated good pain relief and great patient satisfaction.^25^ Subsequently to this pilot, the ED launched an educational initiative of roughly 12 sessions (30 hours) for physicians and nurses prior to full implementation of the opioid reduction protocols (Appendix 1 and 2).

**Population Health Research Capsule**

What do we already know about this issue?

Targeted emergency department (ED) clinician and patient education on minimizing opioid use in favor of non-opioid analgesics is associated with significant reduction in total opioid prescriptions.

What was the research question?

Would there be a change in the patterns of analgesic prescribing for ED patients with renal colic before and after an opioid reduction initiative?

What was the major finding of the study?

An opioid reduction initiative resulted in a reduction in opioid administration in the ED by 12.7% and at discharge by 25.5%.

How does this improve population health?

Similar initiatives in EDs across the United States might reduce opioid administration for ED patients with renal colic and decrease opioid prescribing at discharge.

Patients enrolled in the study were divided into three periods (phases) based on the inception and implementation of an opioid reduction initiative: 2012–2014 (pre-implementation phase); 2014–2015 (implementation phase); and 2015–2017 (post-implementation). The data obtained included the following: age; gender; chief complaints of abdominal and flank pain; final diagnoses of renal colic, kidney stone, nephrolithiasis, urinary calculus, and calculus in the kidney; and analgesics administered in the ED (primary and rescue) and at discharge with name, dose, route and frequency of administration. Two non-blinded abstractors (AT and MB) independently reviewed patients’ EMRs and retrieved data on pain scores, analgesics administered (primary and rescue) with their respected dosing, route, and frequency of administration in the ED and at discharge. We entered the data into a Microsoft Excel data abstraction spreadsheet.

The Excel data abstraction spreadsheet created by the principal investigator (PI) has been used for previously conducted, similar research projects. The PI (SM) holds an MD degree and is an expert in the field of EM and data abstraction via EMR. The PI trained all three abstractors on data gathering and entry specific for this study. One of the study investigators (JD) had over five years of experience in abstracting and recording data from ED EMR. JD and SM oversaw all the data abstraction. JD abstracted all the data independently of two primary abstractors (AT and MB) and, in case of discrepancy between two primary abstractors, JD re-reviewed the charts along with SM and reconciled all discrepancies. We conducted this study at a 711-bed urban, community teaching hospital with an annual ED patient census of greater than 120,000 visits. The study was approved by the hospital’s institutional review board.

### Selection of Participants

Patients considered for inclusion were adults aged 18 and older who presented to the ED with a chief complaint of abdominal and flank pain and final diagnoses of renal colic, kidney stone, nephrolithiasis, urinary calculus, and calculus in the kidney.

### Statistical Methods, Data Analysis, Outcome Measures

We imported the Excel data set into the Statistical Analysis System (SAS). We divided time into three phases: pre-implementation phase (September 9, 2012—August 31, 2014), implementation phase (September 1, 2014—August 31, 2015) and post-implementation phase (September 1, 2015—December 30, 2017). We divided analgesics administered to the patients into opioids (morphine, hydromorphone, hydrocodone, hydrocodone/acetaminophen, fentanyl, methadone, oxycodone, oxycodone/acetaminophen, tramadol, meperidine) and non-opioids (acetaminophen, gabapentin, ibuprofen, ketamine, ketorolac, lidocaine, naproxen, ibuprofen). In addition, we distinguished three ordering contexts: 1) orders for discharge medication; 2) orders for medication to be administered within the ED; and 3) orders for rescue medication. In each context, we described each patient as a) taking both opioids and non-opioids, b) taking only opioids, c) taking only non-opioids, or d) taking neither.

We calculated descriptive statistics for the age and sex of ED patients. We did logistic regression analysis of the probability of prescription of the different type of drug based on patient age, sex and time periods, separately for each context. Additionally, we described which particular medications from the above list were never given in each context and tabularized time periods and class of medication used.

## RESULTS

### Sample Description

A total of 4,490 patients were enrolled in the study over the five–year period, of which 3,793 received analgesics in the ED (1,746 patients in pre-implementation phase, 823 patients during an implementation phase, and 1,224 patients in post-implementation phase). At discharge, 3,533 patients received prescriptions for pain medications (1,716 patients in pre-implementation phase, 804 during an implementation phase, and 1,013 patients in post-implementation phase). Patient demographic data are presented in Table 1.

**Figure 1:**
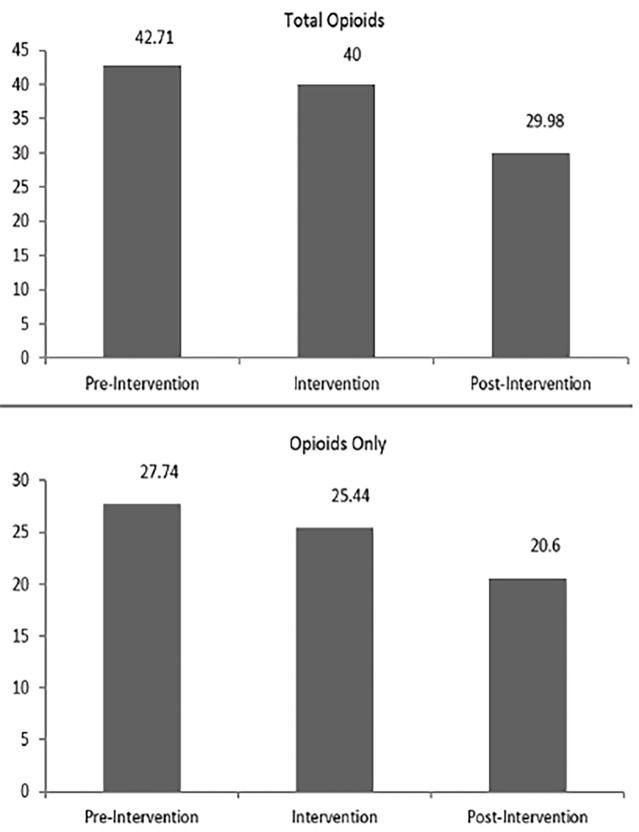
Percentages of opioid analgesic administration in the emergency department.

We observed a meaningful decline in the percentage of patients receiving opioid medications in the ED between pre-implementation and implementation phases (2.71%) and pre-implementation and post-implementations phases (12.73%, 95% confidence interval [CI] [9.56–15.91]; p<0.0001). Similarly, we noted a reduction in opioid administration as primary analgesics between pre-implementation and implementation phases (2.3%) and pre-implementation and post-implementations phases (7.14% 95% CI [1.05–6.46]; p=0.16) (Figure 1).

**Figure 2:**
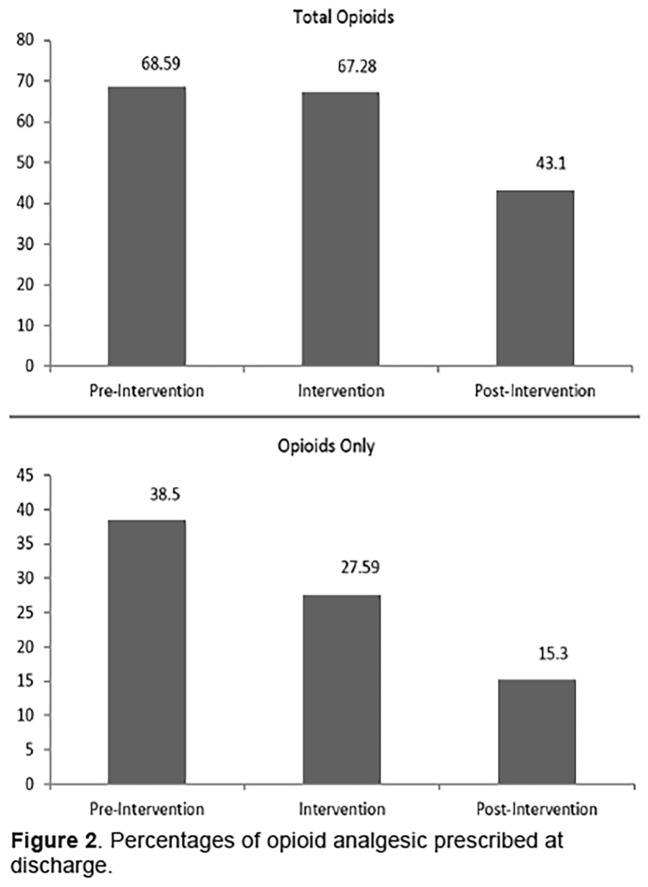
Percentages of opioid analgesic prescribed at discharge.

Furthermore, we saw a significant decrease in opioid administration as rescue analgesics (12.5% 95% CI [9.45–15.05]) between pre- and post-implementation phases. At discharge, we observed a significant decrease in total prescriptions of opioid analgesics (25.49% difference, 95% CI [22.26–28.72]; p<0.0001) and only opioid prescriptions (23.2% difference) between pre-implementation and post-implementation phases (Figure 2).

**Figure 3:**
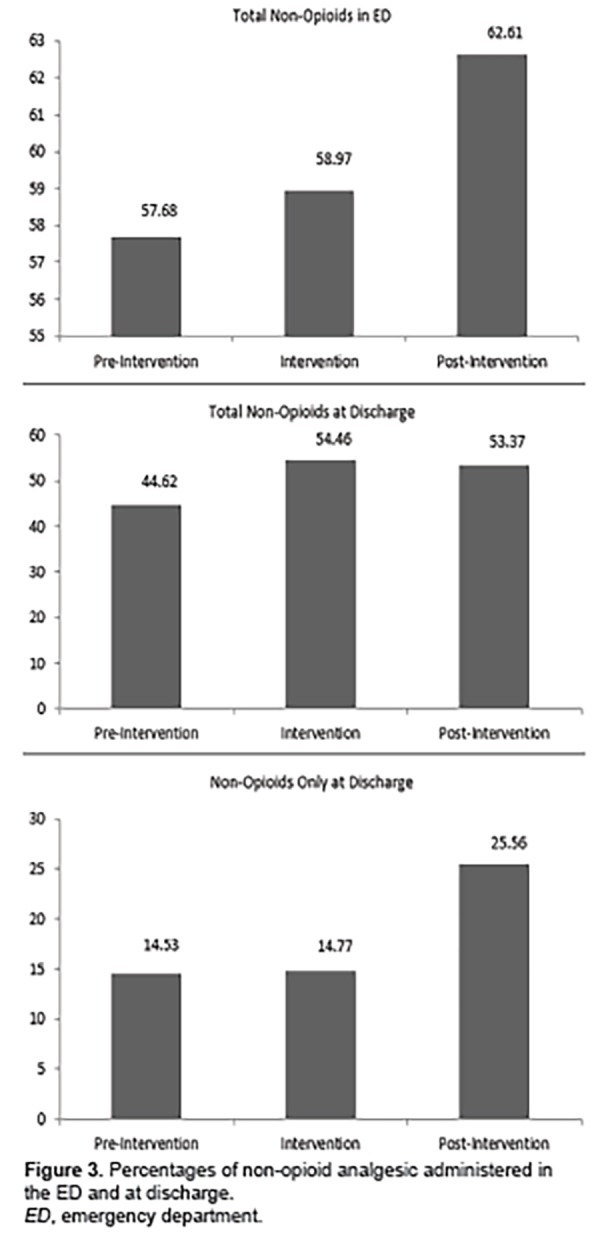
Percentages of non-opioid analgesic administered in the ED and at discharge. *ED*, emergency department.

In addition, we noted an increase in percentage of patients receiving non-opioid analgesics in the ED between pre- and post-implementation phases (4.9% difference) and at discharge (8.75% difference). Similarly, we found a significant increase in non-opioid analgesia at discharge as a sole pain medication from pre-implementation phase to post-implementation phase (11.03% difference) (Figure 3). Data on utilization of specific analgesics (opioids and non-opioids) are presented in Tables 2 and 3.

We observed the largest decrease in administration of parenteral morphine (11.23%) (95% CI [8.1–14.36]; p<0.0001), hydromorphone (0.76%) (95% CI [0.35–1.18]; p=0.0003), and oral oxycodone/acetaminophen (1.29%) (95% CI [0.05–1.7]; p=0.038) between pre- and post-implementation phases. At discharge, we observed the largest decrease in oxycodone/acetaminophen administration (24.69%) (95% CI [21.45–27.94]; p<0.0001) and hydrocodone/acetaminophen between pre and post-implementation phases (5.95% 95% CI [4.69–7.21]; p<0.0001). At the same time we noted an increase in prescribing of morphine sulfate immediate- release tablets at discharge (5.25%) (95% CI [4.1–6.4]; p<0.0001) between pre- and post-implementation phases. We saw an increase in parenteral lidocaine use (0.75%) (95% CI [0.31–1.19]; p=0.0009) as well as an increase in oral acetaminophen (3.23%) (95% CI [2.09–4.36]; p<0.0001) and ibuprofen (1.55%) (95% CI [0.59–2.5]; p=0.0015) in the ED; and acetaminophen (3.21%) (95% CI [2.3–4.13]; p<0.0001) and naproxen (1.1%) (95% CI [0.04–2.17]; p=0.043) at discharge. Lastly, we noted a significant decrease in parenteral morphine (7.84%) (95% CI [5.23–10.44]; p<0.0001) and hydromorphone (0.98%) (95% CI [0.4–1.56]); p=0.0009) rescue administration but an increase in fentanyl rescue (0.51%) (95% CI [0.09–0.93]; p=0.0167) between pre-and post-implementation phases.

## DISCUSSION

We implemented a longitudinal educational program in our ED beginning in 2014 that focused on non-opioid analgesic modalities based on the CERTA approach. This program included a complaint-based, non-opioid medication selection tool (Appendix 1) made available to physicians at the point of patient’s care. We posited that implementing guidelines that promote non-opioid analgesics as a first-line, pain management strategy whenever practicable and appropriate would result in a reduction of opioid prescription in and from the ED.

The results of our study demonstrated that implementation of an opioid reduction protocol in our ED for patients presenting with renal colic decreased the rates of both parenteral and enteral (oral) opioid administration in the ED and at discharge by 12.8% and 25.5% respectively. Consequently to opioid decrease, we observed an increase in non-opioid analgesic utilization by 4.9% in the ED and by 8.75 % at discharge. Perhaps more importantly, two highly addictive opioid analgesics, hydromorphone and oxycodone/acetaminophen combinations (Percocet), had the largest decline in prescribing in the ED and at discharge: 76% decrease in the ED for hydromorphone, and 91% decrease in the ED and 25% at discharge for oxycodone/acetaminophen. Similarly, we observed a decrease in prescribing of opioids as rescue analgesics between pre- and post-implementation phases: 7.8% decrease for morphine, and 98% for hydromorphone.

Of note, we saw a 525% increase in prescribing of morphine sulfate immediate release (MSIR) between pre-and post-implementation phases with a 595% simultaneous decrease in prescribing of hydrocodone/acetaminophen combination. The increase in utilization of MSIR and simultaneous decrease in oxycodone/acetaminophen and hydrocodone/acetaminophen prescribing were largely attributed to departmental safe and judicious opioid prescribing practices geared towards administration of less euphoric opioids in the ED and at discharge.^26^,^27^

In parallel to reduction in opioid prescribing in the ED and at discharge, we observed a significant increase in administration of parenteral lidocaine by 100% during an implementation phase and by 75% in post-implementation phase. We attribute this increase to the departmental implementation of CERTA concept with specific emphasis on intravenous (IV) lidocaine as a viable alternative to opioids in patients with renal colic.^28^,^29^ Additionally, we saw an increase by 384% of oral acetaminophen administration in the ED and by 321% at discharge, as well as 110% increase in naproxen administration at discharge. It is important to emphasize that the results of our study with overall decrease in ED opioid prescribing were not related to opioid shortages during the study periods.

The two biggest challenges faced by authors during the implementation phase of the opioid reduction initiative included lack of familiarity with some of the non-opioid analgesics in the ED (IV lidocaine, sub-dissociative dose ketamine) among physicians and nurses, as well as reluctance to change established practices of pain management among several faculty physicians. Thus, to get a full departmental buy-in of physicians and nurses, a significant amount of time was devoted to the educational piece (opioid and non-opioid analgesics, regulatory and administrative concerns) and to development of non-opioid analgesic protocols focusing primarily on analgesic safety. We believe that addressing these challenges and subsequently getting the full support of the physicians and nurses were the keys to our success in establishing the opioid reduction initiative in our ED and, taken further, might serve as a model for other EDs across the country.

We believe that the concept of patient-specific, pain syndrome-targeted (e.g., renal colic) analgesic therapy can be applied by clinicians to reduce opioid use in EDs across the country. This concept enhances the analgesic armamentarium of emergency physicians and allows broader utilization of non-opioid analgesics. We developed and implemented an opioid reduction initiative that focused on this concept and employed continuous, longitudinal education on strategies to reduce opioid prescribing, promote safe opioid prescribing practices, and encouraged the involvement of patients in shared decision-making about analgesic choices in the ED. Our future research projects are geared toward expanding the role of non-opioid analgesics beyond the ED by creating interdepartmental collaborations and educational initiatives.

## LIMITATIONS

Our study was limited by its retrospective design. Data regarding prescribing information when extracted from EMRs may not always be accurate. In addition, we could not assess or display any data with respect to analgesic efficacy of monotherapy or combinations of analgesics that were used to treat renal colic in the ED. Similarly, we could not evaluate the safety of single agents and their combinations with respect to side effects. Lack of a control group severely limited our ability to conclude that the results of this retrospective project were solely attributable to the opioid reduction initiative and not to other factors operating during the same time period. Lastly, lack of blinding among the abstractors (who were in fact authors) to the study hypothesis may have introduced potential, unintentional bias. Additionally, our study was a single-site study, which may not be generalizable to other EDs.

## CONCLUSION

The opioid reduction initiative resulted in a 12.8% reduction in opioid administration in the ED and 25.5% reduction in opioid prescriptions at discharge over the five-year period. Adoption of similar initiatives in EDs throughout the country has the potential to reduce opioid administration to ED patients who present with renal colic and to impact the opioid epidemic by reducing opioid prescriptions, which are known to lead to recurrent opioid use and abuse.

## Supplementary Information





## Figures and Tables

**Table 1:** Demographics of patients enrolled in study of patients receiving opioid medications for renal colic.

Demographics	Pre-intervention	Intervention	Post-intervention
Mean patient age (SD)	45.8 (14.9)	45.7 (14.6)	49.4 (15.7) P<0.0001
Sex (female)	32.14%	32.41%	32.43% P=0.97

*SD,* standard deviation.

**Table 2:** Percentages of patients receiving individual opioid analgesics.

Analgesics	Pre-intervention	Intervention	Post-intervention
In emergency department			
Morphine sulfate	39.58	36.62	28.35
Hydromorphone	0.83	0.72	0.07
Meperidine	0.05	0	0
Fentanyl	0.05	0	0.27
Oxycodone/acetaminophen	2.2	2.67	1.29
Codeine/acetaminophen	0.15	0	0
At discharge			
Morphine sulfate immediate release	0.05	0.2	5.3
Hydromorphone	0.1	0.1	0
Meperidine	0.05	0	0
Hydrocodone/acetaminophen	7.24	1.44	1.29
Oxycodone	0.2	0.1	0.2
Oxycodone/acetaminophen	61.2	65.74	36.51
Codeine/acetaminophen	1.17	1.23	1.29
Rescue			
Morphine sulfate	23.34	19.79	15.5
Hydromorphone	1.32	2.46	0.34
Meperidine	0.05	0	0
Fentanyl	0.1	0.41	0.61
Oxycodone/acetaminophen	5.77	3.79	1.63
Codeine/acetaminophen	0.1	0	0.07

**Table 3:** Percentages of patients receiving individual non-opioid analgesics.

Analgesics	Pre-intervention	Intervention	Post-intervention
In emergency department			
Ketorolac	55.28	56.51	56.15
Ketamine	0.05	0	0.07
Acetaminophen	1.13	1.33	4.35
Ibuprofen	1.17	0.72	2.72
Lidocaine	0	1.13	0.75
At discharge			
Gabapentin	0	0	0.07
Ketorolac	7	1.33	5.91
Acetaminophen	0.05	0.1	3.26
Ibuprofen	34.74	51.69	42.49
Lidocaine patch	0	0	0.27
Naproxen	1.96	0.51	3.06
Rescue			
Ketorolac	14.33	12.82	10.4
Ketamine	0	0	0.14
Acetaminophen	0.24	0.21	8.33
Ibuprofen	0.59	0.62	0.54
