# Supplementary material for: Analgesic Administration for Patients with Renal Colic in the Emergency Department Before and After Implementation of an Opioid Reduction Initiative
Source: West J Emerg Med. 2018 Oct 18;19(6):1028–35. doi: 10.5811/westjem.2018.9.38875 (PMC6225949; doi:10.5811/westjem.2018.9.38875)
Supplement: Supplementary file 1 [file wjem-19-1028-s001.docx]

**Appendix 1. Channels/Enzymes/Receptors Targeted Analgesia in the ED**

| **Target** | **Drug** | **Indications** | **Pain Syndrome** |
| --- | --- | --- | --- |
| **Sodium Channels Blockers**  **Local Anesthetics**  **Antidepressants:** | **Lidocaine**:  Topical: 5% Lidocaine patch (Lidoderm) (q12h)  Lidocaine 4% patch (q12h)  Local:1-2% (4mg/kg max);with Epi (7mg/kg)  Regional: 1-2% (4mg/kg max)  Intra-articular: 1% (20-30 ml)  Systemic (IV): Preservative –free Lidocaine 2% (1.5-2mg/kg, max 200mg)  **Bupivacaine**:  Local -0.25-0.5% (2.5mg/kg max); 3.5 mg/kg with epi  Regional: 0.25-0.5% (2.5 mg/kg max)  **Nortriptyline**: 25mg po daily  **Amitriptyline**: 10mg po daily | Chronic/Acute MSK Pain  Acute MSK pain  Acute MSK pain  Acute Visceral Pain  Acute MSK Pain  Acute Neuropathic Pain  Chronic Neuropathic Pain  Acute MSK Pain  Acute MSK Pain  Chronic neuropathic pain | Tendinitis, Osteoarthritis, Contusion  Traumatic Injuries  Traumatic Injuries (fractures, dislocations)  Dislocations (shoulder)  Renal Colic  Acute Back Pain  Acute Herpetic Neuralgia  Post-herpetic Neuralgia, Trigeminal Neuralgia.  Traumatic Injuries (lacerations)  Traumatic Injuries (fractures, dislocations, subluxations)  Post-herpetic Neuralgia, Sciatica |
| **Calcium Channels (central) Blockers** | **Gabapentin**: 100-300 mg  **Pregabalin**: 25mg po | Acute Post-operative Pain  Acute Neuropathic pain  Chronic Neuropathic Pain | Nerve Palsies, Neuralgias  Diabetic Neuropathy,  Post-herpetic Neuropathy, Sciatica, Fibromyalgia |
| **Cox-1, Cox-2 Enzymes Inhibitors** | **NSAIDs**:  **Ibuprofen**: 400mg po (1200mg/24h  **Naproxen**: 500 mg po (1g /24h)  **Indomethacin**: 25-50 mg po  **Ketorolac:**  IV: 10-15mg  IM: 30 mg (strongly discouraged)  PO: 10mg (rarely)  Topical:  **Diclofenac** 1% Gel  **Diclofenac** 1.3% Patch  **Acetaminophen:**  PO: 500mg (1500mg/24h for analgesia)  IV: 1g over 15 min q 6h  PR: 650 mg -1300 mg as a single dose | Acute MSK Pain (trauma), Headache, Inflammatory Pain, Chronic MSK pain  Inflammatory Pain  Acute Visceral Pain, Acute MSK Pain  Acute MSK Pain, Acute Visceral Pain  Acute MSK Pain  Acute/Chronic MSK  Acute MSK Pain  Headache  Acute MSK Pain  Acute Visceral Pain (as adjunct)  Headache | Sprains, Strains, Contusions,  Osteoarthritis, Rheumatoid Arthritis Tendinopathies  Gout  Renal Colic, Abdominal Pain (non-traumatic), Back Pain, Headache  Sprains, Strains, Contusions,  Tendinopathies  Sprains, Strains, Contusions,  Tendinopathies, Arthralgias  Sprains, Strains, Contusions,  Tendinopathies, Arthralgias  Sprains, Strains, Contusions,  Tendinopathies, Arthralgias |
| **Central Alpha 1,2 Receptors Agonists** | **Clonidine** IV:  0.15-5µg/kg single dose  0.3 µg/kg/hr continuous infusion  **Dexemedetomidine** IV:  - 0.5-1µg/kg bolus  -0.1-0.5µg/kg/hr infusion | Acute Pain, Chronic Pain (neuropathic pain)  Acute Pain, Neuropathic pain, Opioid-tolerant pain | Adjunct to Local Anesthetics, Opioids, Ketamine for Acute Traumatic/Non-Traumatic Pain  Sickle Cell Pain,CRPS, Sciatica |
| **D1-2 Receptors Antagonists** | **Haloperidol** IV: 1-5mg  **Droperidol** IV: 2-5mg  **Metoclopramide** IV:  10-20mg  **Prochlorperazine** IV:10 mg  **Chlorpromazine** IV:  12.5 mg IV | Acute Pain, Chronic Pain | Migraine Headache, Chronic Abdominal Pain |
| **GABA Receptors Agonist/NMDA Antagonist** | **Propofol** IV:  10mg Q5 min titration  1 mg/kg full PSA dose | Refractory Headache | Intractable Migraine Headache |
| **5HT-2, 5HT-3 Receptors Antagonists** | **Metoclopramide** IV:  10-20mg  **Haldol** IV: 1-5mg  **Droperidol** IV:2-5mg | Acute Pain | Migraine Headache  Tension Headache |
| **5HT-1 Agonists** | **Sumatriptan** SQ:  4-6 mg  IN: 5-20mg, repeat after 2h, max 40mg daily | Acute Pain | Migraine Headache  Cluster Headache |
| **NMDA/Glutamate Receptors Antagonists** | **Ketamine (sub-dissociative dosing):**  IV bolus: 0.1-0.3 mg/kg over 15 min  IV infusion: 0.15-0.25mg/kg/hr titrate q 30min by 2.5-5 mg  IN: 0.5-1mg/kg titrates q 10 min  SQ: 0.1-0.3 mg/kg over 15 min  SQ infusion: 0.15-0.25mg/kg/hr titrate q 30min by 2.5-5 mg  **Magnesium:** 1-2 mg IV over 30-60 min | Acute Pain, Opioid-tolerant pain, Chronic pain (non-cancer, neuropathic, central), Opiod-induced hyperalgesia, Malignant Pain  Acute Pain, Chronic Pain (adjunct to Ketamine) | Acute Traumatic Pain, Abdominal/Flank/Back Pain, Sickle Cell VOC Pain, Sciatica,  Abdominal Migraine, Neuropathic Pain, Refractory Migraine.  Migraine Headache, Cluster Headache (acute/chronic) |
| **Opioid Receptors Agonists (Mu-receptors**) | **Morphine**: (IV, SQ, Nebulization, PCA) weight -based, fixed:  0.1 mg/kg, 4mg  **Hydromorphone** (IV, IM, SQ, PCA) weight-based, fixed:  0.0075-0.015mg/kg, 0.2-1mg  **Fentanyl** (IV, IN, Nebulization, PCA): weight-based, fixed:  0.5 -1µg /kg, 25-50µg, IN: 1-2 µg /kg,  Nebulization: 2-4 µg /kg | Acute Traumatic/Non-traumatic Pain | Acute MSK Pain (fractures), Acute Visceral Pain (Abdominal Pain, Renal colic, Biliary colic), Acute Traumatic/Non-traumatic Pain, Sickle Cell VOC Pain |
| **TRPV1 Receptor Agonists** | **Acetaminophen:**  PO: 500mg (1500mg/24h for analgesia)  IV: 1g over 15 min q 6h  PR: 650 mg -1300 mg as a single dose  **Capsaicin:**  Topical: 0.025-0.15% (gels, creams, lotions, patches)  Topical 8% patch ( Rx only) | Acute MSK Pain  Headache  Acute MSK Pain  Acute Visceral Pain (as adjunct)  Headache  Acute MSK Pain  Chronic Neuropathic pain | Sprains, Strains, Contusions,  Tendinopathies, Arthralgias  Sprains, Strains, Contusions,  Tendinopathies, Arthralgias  Strain, Sprain, Ecchymoses, Arthralgias  PHN, PDN |
| **Volatile Anesthetic (Endogenous Opioid Receptors Agonist)** | **Nitrous Oxide**:  50/50 concentration  70/30 concentration | Acute pain-Traumatic/Non-traumatic | Fractures, Dislocations, Adjunct to Local/Regional Blocks, Opioids |
