# Supplementary material for: Analgesic Administration for Patients with Renal Colic in the Emergency Department Before and After Implementation of an Opioid Reduction Initiative
Source: West J Emerg Med. 2018 Oct 18;19(6):1028–35. doi: 10.5811/westjem.2018.9.38875 (PMC6225949; doi:10.5811/westjem.2018.9.38875)
Supplement: Supplementary file 2 [file wjem-19-1028-s002.docx]

**Appendix 2. Non-Opioid Analgesic Modalities in the ED**

| **Pain syndrome** | **Inpatient (in the ED) parenteral opioid alternatives** | **Outpatient (discharge) oral opioid alternatives** |
| --- | --- | --- |
| **Abdominal Pain (non-traumatic)** | IV Ketorolac: 10-15 mg IVP  IV Lidocaine: 1.5 mg/kg of 2% (preservative-free Lidocaine) over 10-15 min (max dose 200 mg)  IV Acetaminophen (APAP): 1g over 15 min (as adjunct if NPO or contraindications to NSAID’s, Lidocaine or Ketamine)  IV Ketamine (SDK): 0.3 mg/kg over 10 min, + IV infusion at 0.15 mg/kg/hr | PO Acetaminophen: 500 mg po q8h (max 1500mg/day). Best if combined with Ibuprofen at 400mg q8h  PO Ibuprofen: 400 mg q8h x 3 days (max 5 days with 1200 mg/d) |
| **Abdominal Pain (traumatic)** | IV Acetaminophen: 1g over 15 min (as adjunct if NPO or contraindications to NSAID’s, Lidocaine or Ketamine)  IV Ketamine (SDK): 0.3 mg/kg over 10 min, + IV infusion at 0.15 mg/kg/hr  IV Lidocaine: 1.5 mg/kg of 2% Lidocaine (preservative free) over 10-15 min (max dose 200 mg)  Transverse Abdominis Plane Block (abdominal wall hematoma, superficial lacerations) with Lidocaine 1%-2%, Bupivacaine 0.5% or Chloroprocaine 2% | PO Acetaminophen: 500 mg po q8h (max 1500mg/day). Best if combined with Ibuprofen at 400mg q8h  PO Ibuprofen: 400 mg q8h x 3 days (max 5 days with 1200 mg/d)  Topical NSAID’s (Diclofenac Cream, Patch) for abdominal wall trauma  Topical Lidocaine (4-5% patch, 2% cream) for abdominal wall trauma  Topical Capsaicin (0.025-0.15% cream) for abdominal wall trauma |
| **Back Pain (non-radicular)** | IV Ketorolac:10-15mg IVP (non-penetrating trauma)  IV Lidocaine:1.5 mg/kg of 2% Lidocaine (preservative free) over 10-15 min  IV Acetaminophen: 1g over 15 min (as adjunct if NPO or contraindications to NSAID’s, Lidocaine or Ketamine)  IV Ketamine (SDK):0.3 mg/kg over 10 min, + IV infusion at 0.15 mg/kg/hr  Trigger point injection: up to 10 ml 0.5% Bupivacaine or 10 ml of 1% Lidocaine to site of maximal pain | PO Ibuprofen: 400 mg q8h x 3 days (max 5 days with 1200 mg/d)  PO Acetaminophen: 500mg po q8h (max 1500 mg/day). Best if combined with Ibuprofen at 400mg q8h  Topical Diclofenac Gel/Patch: to affected area q12h for 5-7 days  Topical Lidocaine (4-5% patch, 2% cream)  PO Methocarbamol: 500 mg-1500 mg q6h-8h for 2-3 days  PO Diazepam: 5 mg q8h for 2-3 days  Physical Therapy  Acupuncture when available |
| **Burns** | IV Ketamine (SDK): 0.3 mg/kg over 10 min, + IV infusion at 0.15 mg/kg/hr  IV Magnesium: 1g over 30-45 min (as adjunct to Ketamine)  IV Lidocaine – 1.5 mg/kg of 2% Lidocaine (preservative free) over 10-15 min, + continuous infusion at 1.5.-2.5 mg/kg/hr  IV Dexmedetomidine: 0.25-0.5 mcg/kg/hr infusion  UGRA (Nerve Blocks) of extremities, thorax, neck and abdominal wall with Lidocaine 1%-2%, Bupivacaine 0.5% or Chloroprocaine 2% | Topical Lidocaine 5% cream: to affected area q12h  PO Ibuprofen: 400 mg q8h x 3 days (max 5 days with 1200 mg/d)  PO Acetaminophen: 500mg po q8h (max 1500 mg/day). Best if combined with Ibuprofen at 400mg q8h  Topical Diclofenac gel 1%-q12h for 3-5 days (small areas, first degree) |
| **Headache** | IV Metoclopramide:10 mg (slow infusion over 10-15 min) or  IV Prochlorperazine:10 mg (slow infusion over 10-15 min) with IV Diphenhydramine: 25-50 mg (for akathisia/agitation prophylaxis)  IV Chlorpromazine: 12.5 mg (slow infusion in 500ml over 30min)  IV Ketorolac: 10-15 mgIVP  IV Magnesium: 1-2 gm over 46-60 min  IV Dexamethasone: 10mg IV (migraine recurrence)  SQ Sumatriptan (migraine): 6mg (within 1hr of onset, 12mg 1hr later if needed)  Paracervical injections: Lidocaine 1%-2%, Bupivacaine 0.5%  Occipital Nerve Block: Lidocaine 1-2%, Bupivacaine 0.5 %  Refractory cases:  IV Haldol: 2.5-5 mg IV (slow infusion over 10 min)  IV Propofol (intractable migraine): 10mg IVP q5 min until HA is tolerable  Ketamine (SDK): 0.3mg/kg slow infusion (over 10 min | PO Acetaminophen/Aspirin/Caffeine (Excedrin): 2 caplets q6h-8h  PO Ibuprofen: 400 mg q8h x 3 days (max 5 days with 1200 mg/d)  PO Acetaminophen: 500mg po q8h (max 1500 mg/day). Best if combined with Ibuprofen at 400mg q8h  PO Sumatriptan: 100mg (no more than 200 mg per 24h)  Intranasal (IN) Sumatriptan: 5-20mg, repeat after 2h, max 40mg daily  PO Rizatriptan: 10mg (no more than 20 mg per 24h) |
| **Musculoskeletal Pain (Traumatic/Non-traumatic)** | PO Ibuprofen: 400 mg  PO Acetaminophen: 500mg  Topical Diclofenac Gel 1%  PO Methocarbamol: 500 mg – 1500 mg  UGRA (Nerve blocks) with Lidocaine 1%-2%, Bupivacaine 0.5% or Chloroprocaine  IV Ketorolac – 10 mg IVP  IV Ketamine (SDK): 0.3 mg/kg over 10 min, + IV infusion at 0.15 mg/kg/hr  IV Magnesium: 1g over 30-45 min (as adjunct to Ketamine)  IV Acetaminophen: 1g over 15 min (as adjunct if NPO or contraindications to NSAID’s, Lidocaine or Ketamine) | PO Ibuprofen: 400 mg q8h x 3 days (max 5 days with 1200 mg/d)  PO Acetaminophen: 500mg po q8h (max 1500 mg/day). Best if combined with Ibuprofen at 400mg q8h  Topical Diclofenac Gel/Patch: to affected area q12h for 5-7 days  Topical Lidocaine (4-5% patch, 2% cream): q12 h to affected area for 5-7 days  Topical Capsaicin (0.025-0.15% cream): q 12 h to affected area  PO Methocarbamol: 500 mg – 1500 mg q6h-8h for 2-3 days  Physical Therapy |
| **Neuropathic Pain** | Trigger point injection: up to 10 ml 0.5% Bupivacaine and 10 ml of 1% Lidocaine to site of maximal pain  Lidocaine 5% patch  PO Gabapentin: 300 mg loading dose  IV Ketamine (SDK): 0.3 mg/kg over 10 min, + IV infusion at 0.15 mg/kg/hr  IV Magnesium: 1g over 30-45 min (as adjunct to Ketamine)  IV Lidocaine:1.5 mg/kg of 2% Lidocaine (preservative free) over 10-15 min, + continuous infusion at 1.5-2.5 mg/kg/hr  IV Dexmedetomidine: 0.25-0.5 mcg/kg/hr infusion | PO Ibuprofen: 400 mg q8h x 3 days (max 5 days with 1200 mg/d)  PO Acetaminophen: 500mg po q8h (max 1500 mg/day). Best if combined with Ibuprofen at 400mg q8h  PO Gabapentin: 100 mg q8h (titrate by 100 mg every other day up to 600-900 mg/day)  PO Pregabalin: 25 mg tid (titrate by 25 mg every other day up to 150 mg/day  Topical Lidoderm 4%-5% patch: 2 patches max for 12h on skin, then 12h patch-free period  Topical Capsaicin cream 0.025%, patch 8%: apply q12h (causes severe skin irritation)  Topical Lidocaine cream 2.5%, 3%; gel 2% apply topically q8h |
| **Renal Colic** | IV Ketorolac: 10-15 mg IVP  IV Lidocaine: 1.5 mg/kg of 2% Lidocaine (preservative free) over 10-15 min  IV Acetaminophen: 1g over 15 min (as adjunct if NPO or contraindications to NSAID’s, Lidocaine or Ketamine)  IV Ketamine (SDK): 0.3 mg/kg over 10 min, + IV infusion at 0.15 mg/kg/hr  IN Desmopressin: 40 mcg once as adjunct to NSAID’s  PO Ibuprofen 400mg plus Acetaminophen 500mg ( if patients is able to tolerate po) | PO Ibuprofen: 400 mg q8h x 3 days (max 5 days with 1200 mg/d) or  PO Naproxen: 500 mg q12h x3 days  PO Acetaminophen: 500mg po q8h (max 1500 mg/day). Best if combined with Ibuprofen at 400mg q8h  PO Tamsulosin: 0.4mg daily until stone passage (only for distal ureteral stones >6mm) |
| **Sickle Cell Vaso-Occlusive Painful Crisis** | IN Ketamine (SDK): 1mg/kg (no more than 1ml per nostril) to start  IV Ketamine: 0.3 mg/kg over 10 min, + IV infusion at 0.15 mg/kg/hr  IV Magnesium: 1g over 30-45 min (as adjunct to Ketamine)  IV Haloperidol: 2.5-5mg  IV Dexmedetomidine: 0.25-0.5 mcg/kg/hr infusion  IV Ketorolac: 10-15 mg  SQ Ketamine (SDK) infusion: 0.15-0.25mg/kg/hr if no IV access | PO Ibuprofen: 400 mg q8h x 3 days (max 5 days with 1200 mg/d) or  PO Naproxen: 500 mg q12h x3 days  PO Acetaminophen: 500mg po q8h (max 1500 mg/day). Best if combined with Ibuprofen at 400mg q8h  Physical Therapy  Acupuncture  Transcutaneous Electrical Nerve Stimulation |
